# Editorial: User psychology and behavior regarding healthcare IT

**DOI:** 10.3389/fpsyg.2022.1068517

**Published:** 2022-11-15

**Authors:** Fanbo Meng, Xiaofei Zhang, Libo Liu

**Affiliations:** ^1^School of Business, Jiangnan University, Wuxi, China; ^2^Business School, Nankai University, Tianjin, China; ^3^School of Computing and Information Systems, The University of Melbourne, Melbourne, VIC, Australia

**Keywords:** user psychology, user behavior, healthcare IT, patient research, editorial

In the last decade, it has been proven that healthcare information technology (IT) has significant potential for (1) improving service quality, (2) redistributing healthcare resources, (3) reducing healthcare cost, and (4) alleviating rural-urban health disparities (Agarwal et al., 2010; Goh et al., 2011; Mein Goh et al., 2016). During the COVID-19 pandemic, the public were able to efficiently manage their health conditions, receive social support, and attend remote health consultations through the use of relevant healthcare IT (Wood et al., 2019). Despite these benefits, several challenges are associated with the sustainable use of healthcare IT, including (1) physicians' under-contributions (Kim and Mrotek, 2016), (2) low patient engagement (Mirzaei and Esmaeilzadeh, 2021), and (3) poor membership retention and commitment in online platforms (Xing et al., 2018). Considering that healthcare IT involves personalization and human–computer interactions, researchers' sole focus on investigating factors related to the information technology side of healthcare IT is not sufficient enough to increase our understanding or to address these challenges. Accordingly, the objective of this Research Topic is to call for further investigations on user behavior regarding healthcare IT across multiple disciplines such as psychology, information systems, and human–computer interaction.

This Research Topic on “*User Psychology and Behavior Regarding Healthcare IT*” includes nine articles that address the abovementioned challenges related to the use of healthcare IT. These articles investigate information/knowledge sharing behaviors and usage behaviors, and incorporate (1) psychological rewards, (2) patients' trust, (3) health information privacy concerns, (4) social identity, (5) psychological distances, (6) prosociality, (7) anxiety, and other psychological factors in their research models. They include population samples of physicians, chronic patients, and the general population in the context of online health platforms and mobile health apps. More importantly, these articles offer valuable insights into user psychology and behavior regarding healthcare IT. Below, we summarize five of the nine articles:

Yao and Sheng examine the influence of psychosocial and technological factors on health information sharing adoption in the context of social sharing services. The authors develop a hypothesized model for health information social sharing adoption (HISSA), integrating attitude beliefs, control beliefs, and normative beliefs. The model is empirically tested using a cross-sectional survey of 375 participants from China. The results show that the psychosocial factor normative beliefs is the most critical factor influencing user adoption intent. The proposed model also has practical implications for understanding the influences of these factors on user adoption behavior in a health context.

Tomczyk focuses on the psychometric properties of the German version of the information privacy concern (AIPC) scale, regarding the use of COVID-19 contact-tracing apps. Three-factor and four-factor models are empirically tested using a cross-sectional survey of 349 participants in Germany. The main findings include: (1) all factors in the four-factor model show good reliabilities and convergent as well as discriminant validities. The four-factor model is preferable compared with the three-factor model; (2) health information privacy concern is negatively associated with attitudes as well as use intention regarding contact-tracing mobile apps; and (3) factors measuring anxiety and personal attitude significantly overlap.

Zhang S. et al. attempt to understand the impact of previous first-aid experience on the online learning of first-aid knowledge and skills. Drawing on the construction level and prosociality theories, the authors consider individual psychological factors, and develop a research model for the psychological distances and prosociality mediating roles. The results show that previous first-aid experience positively impacts online first-aid learning intention. In addition, psychological distance from first-aid events, and prosociality, play significant mediating roles in the relationship between first-aid experience and learning intention. This study contributes to understanding first-aid learning intention by revealing the impact of individual psychological factors.

Guiomar et al. investigate the usability of the iACTwithPain platform in patients with chronic diseases and healthcare professionals. In a series of experiments, the authors examine participants' responses toward an intervention (video-animation, real-image video, and videoscribe animated video) while using the iACTwithPain platform. The main findings show that chronic patients prefer real-image video over animations or audio, whereas healthcare professionals are more attracted to the appealing and dynamic aspects of an animation. The intervention can significantly improve patient engagement and retention in the iACTwithPain platform, targeting the chronic patient market.

Zhang X. et al. investigate how patient visits and patient consultations influence physicians' online knowledge sharing, considering the contingent roles of physicians' online expertise and online knowledge sharing experience. Based on 6-month panel data from 45,449 physician-month observations in an online health platform in China, results indicate that both patient visits and patient consultations are positive regarding physicians' online knowledge sharing. Specifically, online expertise weakens the positive effects of patient consultations on physicians' online knowledge sharing. Moreover, the online knowledge sharing experience weakens the positive relationship between patient visits and physicians' online knowledge sharing, and enhances the positive relationship between patient consultations and physicians' online knowledge sharing.

Research on user psychology and behavior, in the context of healthcare IT, is lacking in terms of psychology, information systems, and human–computer interactions. Hence, this Research Topic addresses the gap in the literature by contributing to a better understanding of the influences of user psychology and behavior on the utilization of healthcare IT, and offers practical insights into how healthcare providers can improve the use of their healthcare IT.

## Author contributions

FM: writing—original draft. XZ and LL: review and editing. All authors contributed to the article and approved the submitted version.

## Funding

This study was supported by National Natural Science Foundation of China (72271131 and 72001094), Young Elite Scientist Sponsorship Program by Tianjin (TJSQNTJ-2020-12), and High-End Technology Innovation Think Tank Youth Project of China Association for Science and Technology (2021ZZZLFZB1207087).

## Conflict of interest

The authors declare that the research was conducted in the absence of any commercial or financial relationships that could be construed as a potential conflict of interest.

## Publisher's note

All claims expressed in this article are solely those of the authors and do not necessarily represent those of their affiliated organizations, or those of the publisher, the editors and the reviewers. Any product that may be evaluated in this article, or claim that may be made by its manufacturer, is not guaranteed or endorsed by the publisher.
